# Biting Down on Longevity: Correlating Microhardness, Nanoroughness, and Wear Resistance of Milled vs. 3D-Printed Dental Polymers

**DOI:** 10.3390/polym18151877

**Published:** 2026-07-30

**Authors:** Roxana Diana Vasiliu, Georgiana Osiceanu, Flavia Roxana Bejan, Mihaela Ionela Gherban, Diana Uțu, Sorin Daniel Porojan, Anamaria Matichescu, Liliana Porojan

**Affiliations:** 1 Department of Dental Prostheses Technology (Dental Technology), Centre for Advanced Technologies in Dental Prosthodontics, Faculty of Dental Medicine, “Victor Babes” University of Medicine and Pharmacy Timisoara, Eftimie Murgu Sq. No. 2, 300041 Timisoara, Romania; roxana.vasiliu@umft.ro (R.D.V.); flavia.toma@umft.ro (F.R.B.); sliliana@umft.ro (L.P.); 2 National Institute for Research and Development in Electrochemistry and Condensed Matter, 300569 Timisoara, Romania; mihaelabirdeanu@gmail.com; 3 Department of Pharmacology-Pharmacotherapy, Faculty of Pharmacy, “Victor Babes” University of Medicine and Pharmacy Timisoara, Eftimie Murgu Sq. No. 2, 300041 Timisoara, Romania; diana.utu@umft.ro; 4 Department of Oral Reabilitation (Dental Technique), “Victor Babes” University of Medicine and Pharmacy Timisoara, 300041 Timisoara, Romania; porojan.sorin@umft.ro; 5 Department of Preventive, Community Dentistry and Oral Health, Centre for Advanced Technologies in Dental Prosthodontics, Faculty of Dental Medicine, “Victor Babes” University of Medicine and Pharmacy Timisoara, Eftimie Murgu Sq. No. 2, 300041 Timisoara, Romania; matichescu.anamaria@umft.ro

**Keywords:** 3D-printed resins, CAD/CAM blocks, surface topography, microhardness, hydrothermal ageing, thermocycling, atomic force microscopy, mechanical wear, polymer degradation, polymer-infiltrated ceramic network

## Abstract

The nanoscale surface topography and microhardness of additive and subtractive dental polymers were evaluated following exposure to environmental challenges. The study examined two 3D-printed resins (Saremco and Voco) and two milled CAD/CAM blocks (Vita Enamic and Tetric). Specimens were allocated to control or experimental groups and subjected to hydrothermal ageing (thermocycling), in vitro mechanical wear, or a combined protocol involving wear followed by thermal ageing. Surface microtopography was analysed both quantitatively and qualitatively using atomic force microscopy (AFM), while structural stability was assessed through surface microhardness testing. Statistical significance was determined using matrix comparisons (*p* < 0.05). Milled monolithic blocks demonstrated a dense, uniform baseline topography, whereas 3D-printed resins exhibited structural heterogeneity attributed to their layer-by-layer photocuring process. Saremco maintained polymer network stability under thermal stress (*p* = 0.1878), while Voco was highly susceptible to hydrothermal swelling and early matrix plasticization (*p* = 0.0084). The combined protocol of wear and thermal ageing resulted in advanced structural breakdown in all groups (*p* < 0.001). Industrial subtractive blocks exhibited greater resistance to oral environmental stresses. The ceramic framework of Vita Enamic limited polymer domain collapse, whereas Tetric experienced accelerated inter-layer delamination and embrittlement. The combined protocol of wear followed by thermal ageing resulted in significant and uniform degradation of surface microhardness and topographic roughness in all tested groups. Nevertheless, the additively manufactured resins demonstrated substantial structural integrity and exhibited low volumetric wear rates.

## 1. Introduction

Composite resins are the most widely used dental restorative materials because they support minimally invasive dentistry and offer aesthetic results. Various composite restorations in the oral cavity often experience occlusal contact, leading to wear from ongoing masticatory forces. Ideally, these materials should exhibit wear properties similar to those of natural tooth tissues [1]. Enamel typically loses 0.02–0.04 mm vertically per year under normal conditions [2]. Excessive or accelerated wear leads to pathological surface loss, posing significant challenges for dental practitioners and severely impacting patients’ quality of life.

Composite material wear is influenced by tooth characteristics, material type, cavity size, occlusal physiology, and the nature of opposing teeth [3]. The size, distribution, and volume of reinforcing fillers are key factors in restorative materials. Water absorption weakens composite resin by plasticising the organic matrix and reducing the bond between fillers and the matrix. This leads to structural deterioration and accelerated hydrolytic degradation by salivary enzymes [4,5].

Wear of composite resin restorations is evaluated in both clinical and laboratory settings. Clinically, assessment methods are classified as direct or indirect [6]. Casts are then analysed using qualitative methods, such as the Leinfelder and Moffa–Lugassy scales, or quantitative techniques, including measurements of surface topography, roughness, material loss, and fractal dimension. These parameters are measured using mechanical systems, such as stylus profilometry and atomic force microscopy, or optical systems, including laser-scanning microscopy and white-light optical profilometry.

Despite advances in the mechanical properties of composite resins, wear remains a significant issue, particularly for patients with parafunctional habits such as bruxism [7]. In 2015, approximately 800 million composite resin restorations were placed worldwide, with 80% on posterior teeth [8]. The same study found that at least 5% of posterior restorations failed due to fracture, and 12% exhibited significant wear over 10 years. As a result, about 77 million posterior composite resin restorations are expected to show substantial wear, and roughly 32 million placed in 2015 will require repair or replacement due to fracture within the next decade [9]. Wear resistance is a critical factor in clinical decision-making and in the long-term maintenance of restored vertical dimension. However, data on long-term wear performance remain limited. In vitro studies provide a controlled setting to evaluate wear behaviour under standardised conditions. Comparing the wear of 3D-printed and milled CAM-fabricated provisional restorations may yield clinically relevant insights into their performance and indications [10]. Recent studies focus on characterising wear couples, examining the effects of lubrication and the oral environment, and developing more durable restorative materials that minimise antagonistic tooth wear [11].

Wear is the continuous loss of surface material caused by mechanical interaction between two bodies in motion. This process can be simulated, particularly for attrition, using two-body wear configurations. In vitro studies provide an efficient means to assess the wear behaviour of provisional restorative materials. Selecting clinically relevant wear parameters for in vitro models is essential for meaningful results. Quantification typically involves measuring the worn area and volumetric material loss, while surface profilometry is used to characterise the wear scar topography [12]. For dental materials, this configuration offers advantages over traditional methods such as pin-on-disk setups or chewing simulators, as it produces near-circular wear scars that closely replicate clinically observed wear facets and allows straightforward quantification of wear rate.

Multiple laboratory protocols are used in dentistry to evaluate material wear. Each protocol is grounded in specific tribological principles and yields distinct outcomes, including volumetric loss, surface roughness, and topographic alterations [7,8]. Careful selection and interpretation of these methods are essential to generate clinically relevant data on the wear resistance of modern restorative CAD/CAM materials [13].

ISO/TS 14569-2:2001 [14] provides a standardised laboratory framework for evaluating the wear resistance of dental materials under two- and three-body contact conditions that simulate occlusal interactions between opposing teeth. This specification introduced standardised testing procedures with precisely controlled parameters, including load, speed, environment (water or abrasive slurry), and test duration. Wear is quantitatively assessed by measuring mass loss and/or conducting profilometric analyses of volume and height changes relative to designated reference materials [15]. However, biomechanical wear tests remain unstandardized with respect to antagonist type, applied force, frequency, number of cycles, environmental conditions, and surface polishing both before environmental exposure and before testing. Tooth wear primarily results from tooth-to-tooth contact associated with bruxism, clenching, and parafunctional pressures, occurring at an average rate of 8.1 cycles per hour. This rate corresponds to approximately 70,956 cycles per year, or 5913 cycles per month [16].

Alternative testing methodologies differ in applied load, cycle number and frequency, and testing environment. Although wear testing is not required before dental materials are released to the market, any such evaluations should be supported by appropriate laboratory investigations. Clinically, wear is significant because of its negative effects on the functional integrity and aesthetic appearance of restorations [7].

Surface topography influences both the appearance and texture of restorations. Surface roughness plays a key role in material wear, highlighting the need to control topography. ISO 25178-2:2021 [17] is the first international standard for characterising three-dimensional surface texture, providing a unified framework for specifying, measuring, and verifying 3D topography. The standard defines consistent terminology, symbols, and parameter sets for surface analysisand addresses the measurement and interpretation of surface nanoroughness. Among the height parameters, the arithmetical mean height (Sa) is the average of the absolute deviations from the mean plane. In contrast, the maximum height (Sy) is the vertical distance between the highest and lowest surface points [10]. Atomic force microscopy (AFM) uses a cantilever with a sharp tip to examine surfaces at resolutions below the optical diffraction limit. It is also effective for nano-mechanical measurements and exploration [13]. Profilometric measurements were taken in six randomly selected regions within the central and peripheral wear facets, as well as on polished surfaces.

CAD/CAM-milled materials are produced from pre-polymerised, highly cross-linked PMMA blocks processed under high pressure and temperature. This yields a densely polymerised material with reduced porosity and fewer internal defects. After milling, these materials have a smooth surface that requires minimal finishing and can be easily polished to a high gloss. In contrast, the surface roughness of 3D-printed resins depends heavily on printing parameters and the quality of postprocessing and polishing [14].

CAD/CAM-milled provisional resins are typically made from pre-polymerised materials, yielding a homogeneous microstructure. This results in improved mechanical properties and reduced volumetric wear compared with conventionally polymerised resins for interim restorations. These materials are also valued for their antagonist-friendly properties, as their high surface hardness and superior polishability produce a smooth surface that minimises abrasion of opposing teeth [12]. Consequently, PMMA is widely regarded as the reference material for provisional restorations. It is routinely used as a benchmark in studies evaluating newer 3D-printed resins and other digitally manufactured interim materials [14].

Vat-polymerisation technologies, such as stereolithography (SLA) and digital light processing (DLP), are advancing rapidly. These methods build restorations layer by layer and then perform ultraviolet post-curing. Additively manufactured interim resins have traditionally been viewed as less durable than industrially milled materials. However, recent advancements call for a more detailed evaluation that separates surface degradation from structural wear under complex clinical conditions.

The layer-wise structure and potential for incomplete monomer conversion can produce rougher surfaces and weaker interfaces, which may initiate wear and microdamage [7].

The study aimed to evaluate the wear resistance of different subtractively and additively manufactured dental crown materials, including vertical and volumetric loss, and to assess the correlation between wear and the manufacturing method, postprocessing, and surface topography.

The following null hypotheses were formulated:

**H0:**  *Isolated hydrothermal ageing (thermocycling) does not significantly alter the nanoscale surface of CAD/CAM interim materials.*

**H1:**  *In vitro mechanical wear does not cause significant anisotropic changes or grooving in the surface topography of polished CAD/CAM materials.*

**H2:**  *No significant differences are present in baseline surface topography among the four industrially manufactured CAD/CAM brands (Voco, Saremco, Vita Enamic, and Tetric).*

**H3:**  *Simulated clinical wear and hydrothermal ageing do not result in significant alterations in the surface microhardness of CAD/CAM interim materials.*

## 2. Materials and Methods

### 2.1. Sample Preparation

In this study, four types of dental crown resins were included, as shown in Table 1. From Vita Enamic and Tetric, rectangular samples were milled. From Saremco and Voco, the rectangular samples were printed. The materials evaluated in this study were chosen to represent a broad technological range within digital dentistry, encompassing polymer-infiltrated ceramic networks (Vita Enamic), industrially milled composite blocks (Tetric CAD), and contemporary additive manufacturing or 3D-printing resin formulations (Saremco Crowntec and Voco V-Print c&b temp).

The sample size was calculated using G*Power v3.0.10. Assuming a normal distribution and an effect size of 0.49, the required sample size was computed for α = 0.05 and a power of 0.90. The calculation indicated that eight samples per group were needed to achieve statistical significance. A larger sample increases the credibility of the results and more accurately reflects the genuine variety of materials and structures [18]. Consequently, this study used 40 specimens (n = 10 per material group). Specialised software, Fusion (Autodesk Inc., San Francisco, CA, USA), was used to design rectangular plates measuring 14 × 10 × 1 mm. These plates were fabricated using a DLP 3D printer (Asiga Max UV, Asiga, Sydney, Australia) at a build angle of 0° and a layer thickness of 50 μm. After five minutes of ultrasonic rinsing in isopropyl alcohol, the plates underwent group-specific post-curing with the BB Midi Plus system (MECCATRONICORE, Pergine Valsugana, Italy) utilising triple-frequency technology (365, 405, and 480 nm). Blocks were sectioned to uniform dimensions for subtractive samples. Specimens were polished incrementally with silicon carbide paper up to #2000 grit. All specimens were stored in distilled water at room temperature before testing.

### 2.2. Wear Tests

Material specimens were mounted in a specially designed tribometer testing machine 

On the specimen plates, a 6.35 mm radius zirconia sphere was loaded in normal contact with 30 N and rotated at 30 rpm. The angle between the line from the sphere’s centre to the specimen contact surface and the rotation axis was 37°.

Flat specimens produce distinct, nearly circular wear scars from which wear parameters can be assessed. The 37° angle refers to the angle between the sample surface and the rotation axis. Composite resin samples were tested against spherical zirconia ball antagonists. The mean Vickers hardness of the zirconia balls was 1325.6 GPa. The specimens were subjected to the antagonist at 30 rpm, with a mean load of 30 N. To simulate the oral environment, a 33% glycerin solution was used as a lubricant to mimic saliva. Tests were conducted at room temperature, and glycerin was applied to the surface of all samples. At cumulative test duration t, the sliding distance L across each scar was calculated using Equation (1).

L = 2πRftsinθ(1)

where θ = 37° is the angle between the line from the sphere’s centre to the specimen contact surface and the rotation axis; optical measurements of the scar radii r were taken both parallel and perpendicular to the sliding direction, and average values were calculated. ImageJ (version 1.46, Java) was used to measure the radii of the counterbody and wear scar. Each specimen underwent 5913 cycles, representing one month of clinical use. After the wear simulation experiments, the specimens were ultrasonically cleaned in distilled water for 3 min before examination.

The wear scar morphology was first examined using low-magnification micrographs. Plates with near-circular wear scars were further analysed using optical microscopy at 4× magnification (Leica DM 100; Mannheim, Germany). Wear surface area and volumetric wear data were evaluated.

Scar volumes (V) were calculated using the conformal relation (2) [7].

V = πr^4^/4R (2)

The maximum wear depth (h, μm) was determined using Formula (3), and mean values were calculated [10].

h =R − (R^2^ − r^2^)1/2 (3)

Here, R is the radius of the counter ball, and r is the radius of the wear scar.

Wear rate (WR), representing the rate of material loss, was determined using Formula (4) [19].

WR (μm/1000 cycles) = Maximum depth of wear (μm) × 10^3^/number of cycles (4)

According to Archard’s law [20], the specific wear rate (SWR) was calculated using Formula (5).

SWR = V/PL [×10^−3^ mm^3^/Nm] (5)

Here, V is the volume loss, P is the applied normal load (force), and L is the total sliding distance.

### 2.3. Topographical Analysis of Nanosurfaces via Atomic Force Microscopy (AFM)

A surface profilometer was used to analyse surface morphology and wear patterns after tribological testing. Both two-dimensional and three-dimensional surface profiles, as well as average surface roughness values, were obtained. Sample characterisation was performed using an atomic force microscope in contact mode (Nanosurf Easy Scan 2 Advanced Research, Nanosurf AG, Liestal, Switzerland). The measured parameters included height Sy (nm) and average nanoroughness Sa (nm). AFM analysis produced three-dimensional images of the sample surfaces with a 2.2 µm × 2.2 µm scan area.

### 2.4. Vickers Microhardness Testing

Measurements were conducted at selected points using the DM 8/DM 2 microhardness tester (Yang Yi Technology Co., Ltd., Tainan City 70960, Taiwan) equipped with a digital camera. A diamond pyramidal indenter was applied with a 300 g load for 10 s. Following removal of the indenter, indentation dimensions were recorded under a microscope at 40× magnification. Five measurements were performed on each surface, and mean values were calculated.

### 2.5. Statistical Analysis

Data are reported as means with standard deviations. Differences between materials at each stage were evaluated using unpaired Student’s *t*-tests. To control for the increased risk of Type I error due to multiple pairwise comparisons among the four materials, a Bonferroni correction was implemented. For the six pairwise combinations, the significance threshold was set at *p* = 0.0083 (0.05/6). Paired Student’s *t*-tests were used to compare values before and after wear within each material group, with significance defined as *p* = 0.05. Relationships between variables across phases were analysed using regression analysis and Pearson correlation. Pearson correlation coefficients (r) were categorised as follows: 0 to 0.2 (very weak), 0.2 to 0.4 (weak), 0.4 to 0.6 (moderate), 0.6 to 0.8 (strong), and 0.8 to 1.0 (very strong). Unless Bonferroni-adjusted, statistical significance was defined as *p* = 0.05.

## 3. Results

### 3.1. Wear Test

**Figure 1:**
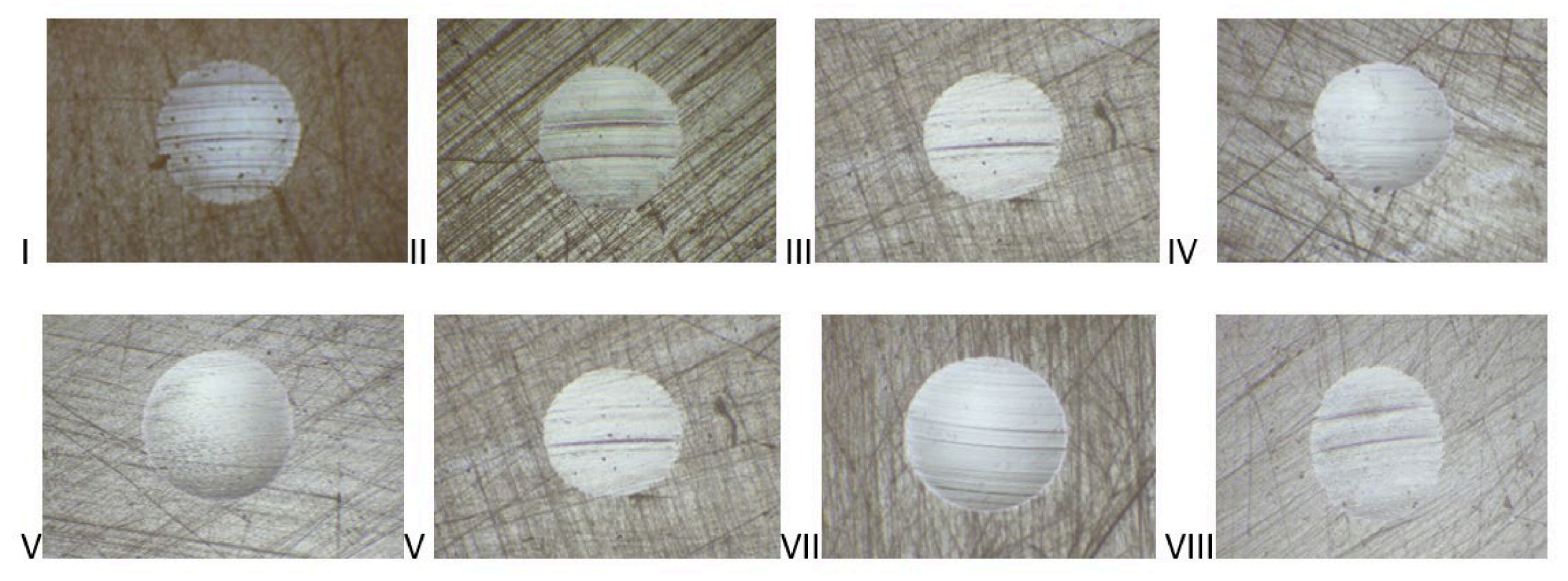
Microscopic images of the wear scars before and after thermal ageing. I: Voco; II: Saremco; III: Vita Enamic; IV: Tetric; V: Voco after thermal ageing; VI: Saremco after thermal ageing; VII: Vita Enamic after thermal ageing; VIII: Tetric after thermal ageing.

This study evaluated the surface stability of four CAD/CAM materials after thermal ageing and two-body wear. Microscopy revealed third-body abrasive wear tracks (200 min, 144 mm) on all samples. Thermal cycling before cumulative wear testing (W + TC) did not increase material degradation. Independent *t*-tests showed a significant reduction or stabilisation in volumetric material loss compared to mechanical wear alone. This unexpected flattening of the wear curve removed the initial material-specific hierarchies seen in baseline testing, indicating structural stabilisation of the polymer networks during thermal exposure. These degradations and the wear scars are shown in Figure 1 and Table 2.

After thermal ageing, Vita Enamic showed the largest reduction, with volumetric loss decreasing to 0.54 ± 0.30 mm^3^ and specific wear rate dropping to 0.35 ± 0.08.

Thermal ageing reduced Tetric’s volumetric loss from 0.48 ± 0.015 to 0.24 ± 0.30 and wear depth to 0.016 ± 0.02. The 3D-printed resins Voco and Saremco showed similar reductions. Volumetric wear loss decreased to 0.170 ± 0.40 mm^3^ for Voco and 0.188 ± 0.15 mm^3^ for Saremco. The wear rate per 1000 cycles declined to 2.45 ± 0.06 for Voco and 2.55 ± 0.50 for Saremco, the lowest among all aged specimens. In summary, the data show that although the milled hybrid ceramic (Vita Enamic) is initially highly susceptible to abrasion, all polymer networks experience a significant physical transition after ageing. This transition reduces both macroscopic volume loss and surface penetration depth under mechanical loading.

### 3.2. Assessment of Nanoroughness

**Figure 2:**
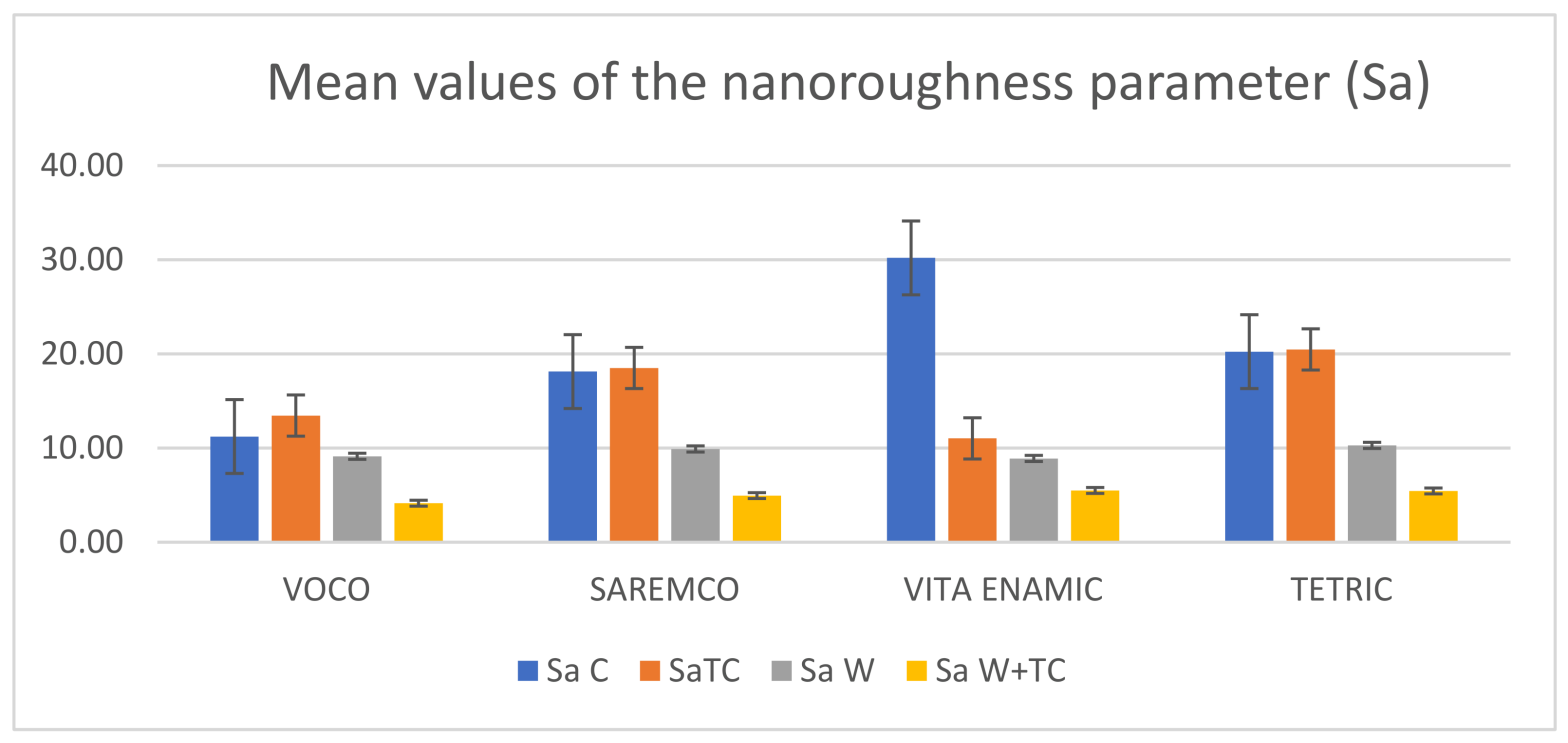
Mean and standard deviation of the nanoroughness parameter (Sa). C—control; TC—thermal ageing; W—wear; W + TC—wear and thermal ageing.

Statistical analysis results are presented in Table 3 and Table 4, and Figure 2. For Saremco (S), thermocycling alone (SC-TC, *p* = 0.1878) does not significantly affect maximum roughness. By contrast, both mechanical polishing (SC-SW, *p* = 0.0105) and combined treatments (SC-SW-TC, *p* = 0.0004) significantly reduce roughness, confirming the effectiveness of mechanical polishing. For Voco (V), thermocycling (VC-TC, *p* = 0.0084) significantly increases roughness, indicating that thermal shock damages the matrix–filler interface. However, combined wear (VC-W-TC, *p* = 0.0001) fully mitigates this damage. For Vita Enamic (VI), thermocycling (VIC-VITC, *p* = 0.0003) significantly reduces roughness, suggesting that hydrothermal ageing refines the surface by removing sharp microfragments produced by milling. For Tetric (T), both mechanical (TC-TW, *p* = 0.0022) and combined treatments (TTC-TW-TC, *p* = 0.000075) significantly improve surface smoothness, demonstrating the high efficiency of these treatments for this nanohybrid composite.

Surface topography, measured by the arithmetic mean height (Sa), changed significantly under environmental and mechanical stresses. The baseline roughness of polished specimens was 18.12 nm. Thermocycling alone did not cause notable degradation, with mean roughness remaining stable at approximately 18.02 nm, confirming the hydrolytic and thermal stability of the polymer–filler interfaces in all tested materials. In contrast, mechanical wear with a zirconia ball reduced Sa to a mean of 9.90 nm, owing to zirconia’s hardness and smoothness, which effectively polished the surface via two-body abrasion. The lowest roughness and highest surface uniformity were achieved with combined wear and thermocycling, with a mean Sa of 4.95 nm (±1.63 nm). This suggests a synergistic effect, as cyclic thermal stresses likely induced microplastic deformation in the resin matrix, thereby allowing the zirconia ball to further polish the surface. The consistent reduction in Sa across all wear groups indicates no filler plucking or micro-interfacial debonding, reflecting a uniform wear rate between the organic and inorganic phases.

Material-specific responses to environmental and mechanical stresses demonstrate the significant influence of internal microstructure on surface topography. For Saremco and the nanohybrid composites Voco and Tetric, mechanical wear with a zirconia ball established a stable two-body abrasion process. The high hardness of the zirconia effectively polished the polymer matrices and reduced micro-asperities, thereby preventing filler plucking, which would otherwise increase Sa values. For Vita Enamic, the polymer-infiltrated ceramic network (PICN) exhibited a ceramic-dominant wear mechanism, with the rigid ceramic matrix resisting the zirconia ball and resulting in surface flattening rather than material loss. When thermal ageing was combined with mechanical stress, all materials achieved their lowest nanoroughness (mean Sa = 4.95 ± 1.63 nm). This synergy suggests that cyclic thermal stresses induced minor softening or plastic flow in the organic phases, allowing the zirconia ball to further polish the surface. The absence of surface degradation in this group highlights the strong interfacial bond stability between silane-treated inorganic fillers and resin matrices across all four materials.

The statistical analysis (unpaired *t*-test, α = 0.05) of the printed and milled samples showed significant differences (*p* ≤ 0.05).

**Figure 3:**
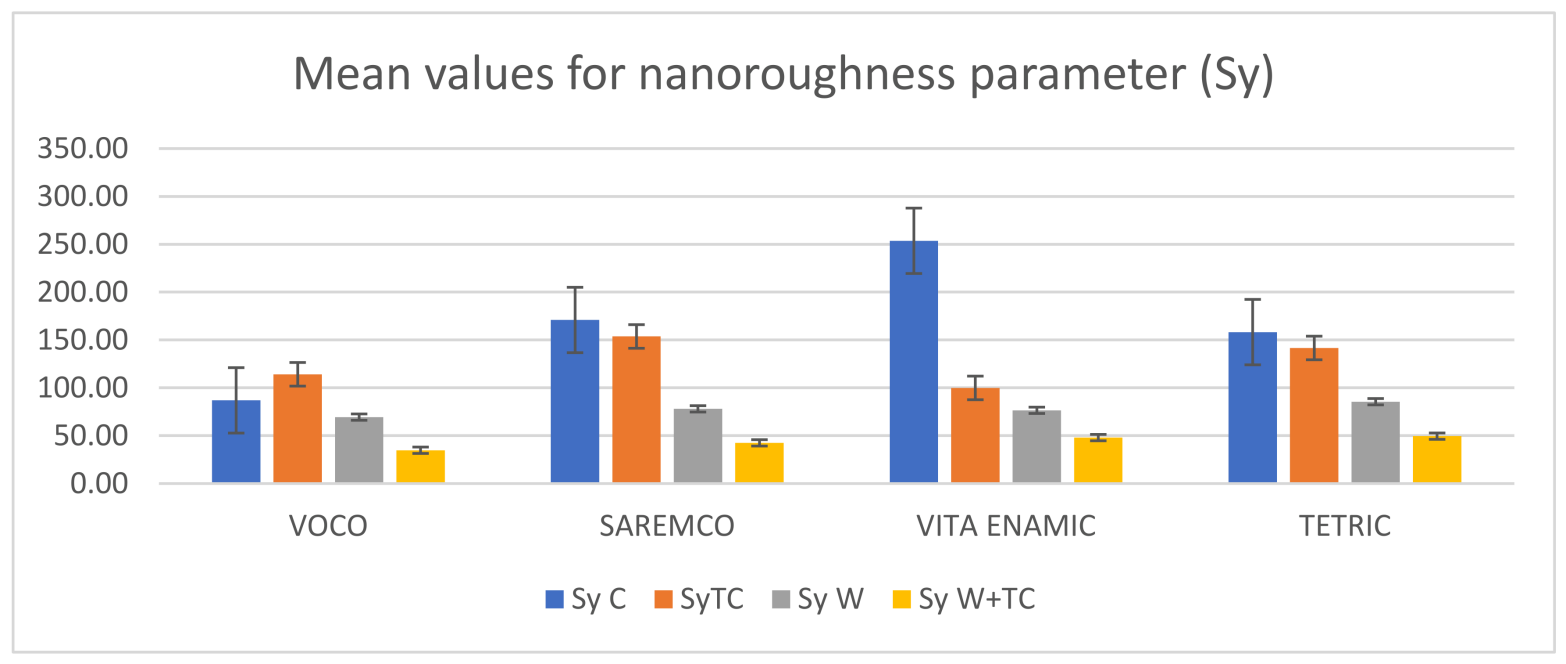
Mean and standard deviation values for the nanoroughness parameter (Sy). C—control; TC—thermal ageing; W—wear; W + TC—wear and thermal ageing.

Assessment of the maximum peak-to-valley roughness height (Sy) provides critical insights into the topographical extremes and structural integrity of dental materials subjected to environmental and mechanical stressors. The informations can be seen in Table 5 and Figure 3. At baseline, the materials displayed markedly different Sy profiles. Voco exhibited the lowest maximum roughness (86.92 ± 25.16 nm), indicating superior immediate polishability and geometric uniformity attributable to its heavily loaded nanohybrid filler composition. In contrast, Vita Enamic showed a substantially higher baseline Sy (253.61 ± 44.37 nm), characteristic of polymer-infiltrated ceramic networks (PICNs) following milling, in which diamond burs induce microfragmentation and deep fissures within the ceramic framework. Following isolated thermocycling (TC), distinct microstructural responses were observed. The Sy value for Vita Enamic decreased significantly to 99.90 ± 17.08 nm, suggesting that hydrothermal cycling promoted the blunting or dissolution of unstable ceramic microfragments remaining from CAD/CAM processing. Conversely, Voco showed an increase in its peak-to-valley threshold (114.15 ± 42.62 nm). As Sy is sensitive to localised defects, this divergence indicates the generation of minor micro-stress at the polymer–filler interfaces, likely due to a mismatch in the coefficients of thermal expansion between the inorganic phase and the resin matrix. Mechanical wear using a smooth zirconia ball functioned as a universal surface-homogenising process. Under isolated wear (W), maximum profile heights were substantially reduced across all groups, converging within a narrow range from 69.41 nm (Voco) to 85.51 nm (Tetric). This consistent reduction reflects a controlled two-body abrasive and burnishing process. The zirconia antagonist effectively removed projecting microasperities without causing subsurface microfractures or interfacial debonding. The flattening effect was further enhanced under the combined regime (W + TC), resulting in all four material classes achieving exceptionally smooth profiles: Voco (34.80 ± 8.67 nm), Saremco (45.52 nm), Vita Enamic (48.01 nm), and Tetric (49.51 nm). The uniform reduction in Sy values under combined thermal–mechanical stress indicates that localised frictional heat and hydrothermal ageing facilitated microplastic flow within the polymer phases. This process enabled the zirconia ball to eliminate residual microvalleys, producing a stable, uniform surface that suggests strong clinical resistance to long-term bacterial colonisation and staining across all evaluated material categories.

**Figure 4:**
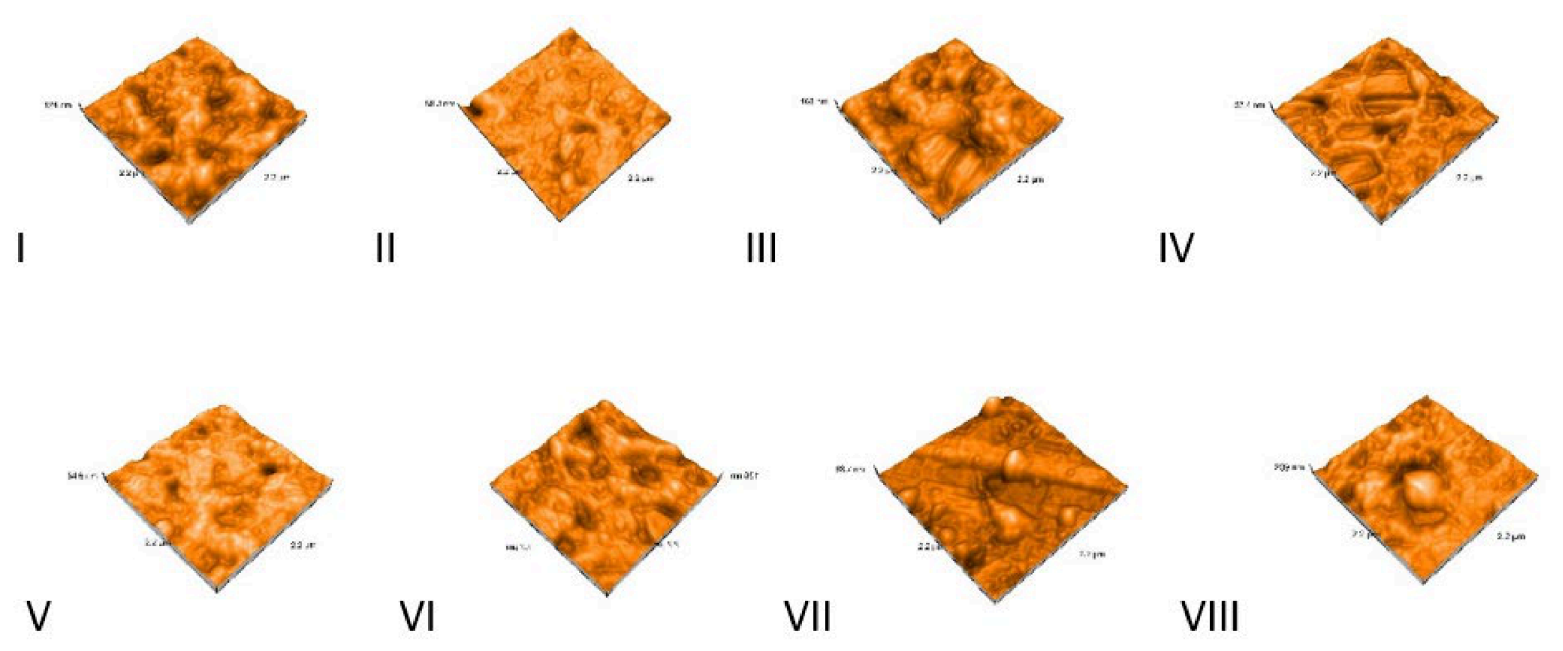
AFM micrographs of the tested samples. I: Voco; II: Saremco; III: Vita Enamic; IV: Tetric; V: Voco after thermal ageing; VI: Saremco after thermal ageing; VII: Vita Enamic after thermal ageing; VIII: Tetric after thermal ageing.

All specimens exhibit distinct surface topographical features within the specified scan zones [e.g., 2.2 × 2.2 µm or 5 × 5 µm]. AFM images show clear changes in surface microtopography following thermal ageing (Figure 4).

The first four groups generally exhibit isotropic or smoothly machined textures before ageing, with gently undulating profiles, minor machining artefacts, and low peak-to-valley amplitudes, reflecting the initial polished state. The surface matrix is uniform and continuous, providing a stable baseline morphology with minimal local height variation.

After thermal ageing in Groups V–VIII, each material develops a more heterogeneous and disrupted topography, indicating matrix degradation, polymer chain scission, or differential thermal expansion. The aged surfaces show higher peaks, deeper valleys, and plateau-like micro-domains, confirming that thermal stress changes the initially uniform texture into an anisotropic, thermally conditioned morphology. Thermal ageing in Group V (Voco) and Group VI (Saremco) results in wider, shallower superficial valleys, with minimal changes in maximum peak height. These results indicate moderate thermo-oxidative degradation and stable resistance to deep structural collapse.

Group VII (Vita Enamic) shows more complex surface restructuring after ageing, with distinct plateau-like ceramic phases and localised resin depletion. These changes suggest differential thermal contraction at the polymer–ceramic interface under prolonged thermal stress.

Group VIII (Tetric) shows higher peaks and deeper, more pronounced microfissures after thermal ageing. These characteristics suggest matrix embrittlement and filler–polymer debonding, indicating a more fragile or heterogeneous microstructure during thermal cycling.

### 3.3. Assessment of Microhardness

**Figure 5:**
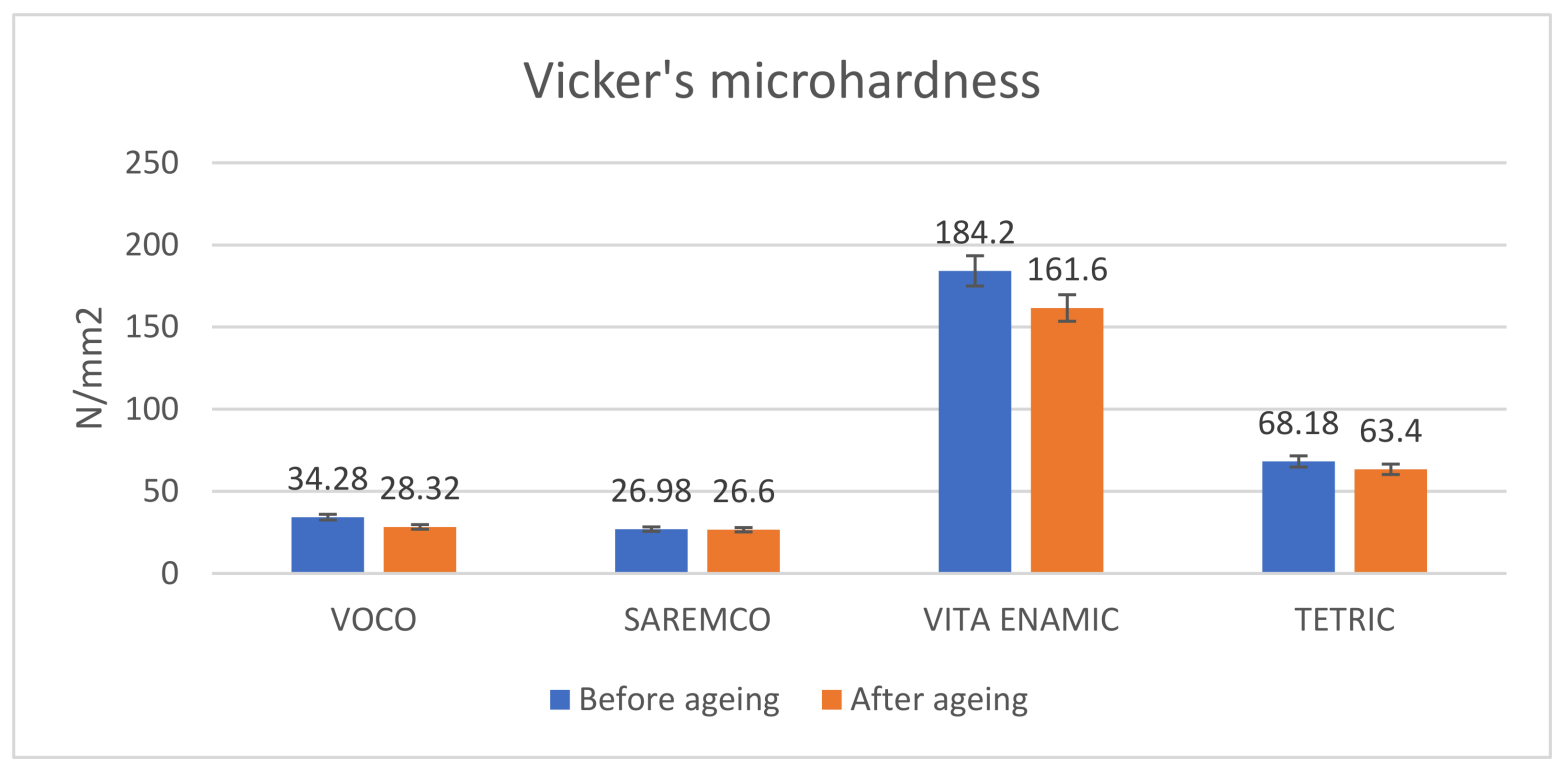
Mean and standard deviation values of Vickers microhardness before and after thermal ageing.

Figure 5 and Table 6 present the Vickers microhardness values (N/mm^2^) for the dental materials before and after thermocycling (TC). Surface hardness ranged from approximately 27 N/mm^2^ to 185 N/mm^2^ across the groups. Before artificial ageing, VITA ENAMIC had the highest mean microhardness (approximately 185 N/mm^2^), followed by TETRIC (approximately 68 N/mm^2^) and VOCO (approximately 35 N/mm^2^). SAREMCO showed the lowest initial value (approximately 27 N/mm^2^). After thermocycling, all groups except SAREMCO showed reduced microhardness: VITA ENAMIC TC decreased to approximately 162 N/mm^2^, TETRIC TC to approximately 63 N/mm^2^, and VOCO TC to approximately 29 N/mm^2^. SAREMCO TC remained stable at about 27 N/mm^2^. The VITA ENAMIC groups exhibited the greatest variance, as shown by the standard deviation error bars. Overlapping error margins between VOCO TC and both SAREMCO subgroups indicate no statistically significant difference between these groups. The effect of artificial ageing by thermocycling (TC) on surface degradation was evaluated using independent Student’s *t*-tests to compare baseline (control) and post-thermocycling values for each dental material. VITA ENAMIC showed a highly significant reduction in surface microhardness after thermocycling (t [8] = 6.694, *p* < 0.001), with mean values decreasing from 184.20 ± 6.42 N/mm^2^ (Control) to 161.60 ± 3.97 N/mm^2^ (TC). In the TETRIC group, thermocycling significantly decreased Vickers microhardness (t [8] = 2.664, *p* = 0.029), from 68.18 ± 2.41 N/mm^2^ to 63.40 ± 3.21 N/mm^2^. The VOCO group experienced a marked reduction in surface microhardness after thermal stress (t [8] = 9.510, *p* < 0.001), dropping from 34.28 ± 0.94 N/mm^2^ (control) to 28.32 ± 1.04 N/mm^2^ (TC). In contrast, SAREMCO maintained high thermal stability, with no significant difference in surface microhardness before and after thermocycling (t [8] = 0.923, *p* = 0.383); mean values were 26.98 ± 0.25 N/mm^2^ (control) and 26.60 ± 0.89 N/mm^2^ (TC).

Hydrothermal ageing and wear significantly reduced the surface microhardness of VITA ENAMIC, TETRIC, and VOCO, with VOCO showing the highest susceptibility to degradation (*t* = 9.510, *p* < 0.001). In contrast, SAREMCO remained stable. These findings indicated that 3D-printed materials, particularly VOCO, experience greater structural softening than milled composites, linking accelerated hydrolytic degradation to matrix plasticization.

## 4. Discussion

The clinical longevity of interim restorations is determined by their surface stability and mechanical integrity in the oral environment. This study systematically evaluated nanoscale surface alterations, as measured by atomic force microscopy (AFM), and microhardness following simulated mechanical wear, hydrothermal ageing, and their combined effects. Inferential statistical analysis indicated that three null hypotheses (H2, H3, and H4) were fully rejected. The null hypothesis (H1) was rejected, as post hoc comparisons showed that in vitro mechanical wear caused only statistically significant anisotropic changes and grooving.

The total rejection of H3 confirms that the investigated materials do not have identical microtopographies in their pristine, control state. This structural variation is statistically supported by significant baseline differences between the 3D-printed Saremco resin and the 3D-printed Voco resin (SC-VC, *p* = 0.0180), as well as between Saremco and the CAM-milled, subtractively manufactured hybrid ceramic Vita Enamic (SC-VIC, *p* = 0.0009).

Although all specimens underwent the same polishing protocols, their baseline nanostructure is determined by their manufacturing technology and chemical composition. Industrial CAD/CAM blocks, such as Vita Enamic and Tetric, are polymerised under optimised high-pressure and high-temperature conditions. This process produces a dense polymer matrix with minimal residual monomer content and maximum polymer–filler conversion [18,19].

In contrast, 3D-printed resins (Saremco and Voco) are built up layer by layer via photopolymerisation. This process yields a lower initial degree of conversion and a reduced cross-linking density prior to post-curing [20]. Interfaces between printed layers may also contain micro-voids or localised polymer heterogeneity. These factors explain why the initial polished surfaces of the printed groups (Saremco vs. Voco) showed statistically distinct baseline textures, in contrast to the uniform, monolithic surfaces of the milled CAD/CAM materials [21,22].

The partial rejection of H1 highlights a key difference in how 3D-printed polymer networks respond to isolated thermal energy. Saremco maintained stable surface topography under thermal stress (SC-TC, *p* = 0.1878), showing minimal sensitivity to temperature changes. In contrast, Voco was highly susceptible to hydrothermal energy (VC-TC, *p* = 0.0084). In 3D-printed resins, thermal exposure increases water sorption, which separates loosely bound polymer chains and acts as a plasticiser. This process accelerates the leaching of unreacted monomers and causes superficial swelling of the polymer. The significant *p*-value for Voco suggests a higher water-uptake rate or a more hydrophilic monomer matrix than for Saremco.

Mechanical wear accelerates degradation across all groups, leading to the complete rejection of H2. Isolated abrasion caused significant changes to the polished surface of Saremco (SC-SW, *p* = 0.0105). Atomic force microscopy (AFM) scans showed a shift from a gently undulating, isotropic texture to an anisotropic topography with directionally structured abrasion marks. Ergistic degradation occurs when thermal ageing and mechanical wear are applied sequentially, resulting in strong rejection of H4.

The results section revealed that both additively and subtractively manufactured materials experienced significant reductions in volumetric loss (V), wear depth (H), specific wear rate (SWR), and wear rate per 1000 cycles (WR) after thermal ageing. Before ageing, Vita Enamic had the highest volumetric loss (2.07 ± 0.03 mm^3^) and specific wear rate (1.06 ± 0.35 × 10^−3^ mm^3^/N⋅m). After ageing, these values decreased to 0.54 ± 0.30 mm^3^ and 0.35 ± 0.08 × 10^−3^ mm^3^/N⋅m, respectively. The 3D-printed resins (Voco and Saremco) and the milled composite (Tetric) also showed improved macro-wear resistance after thermal cycling.

Two mechanisms explain this phenomenon. For the 3D-printed networks (Voco and Saremco), thermal energy from hydrothermal cycling at 55 °C acts as an extended post-curing phase. Light polymerisation in additive manufacturing often leaves residual unreacted monomers in the matrix [23,24]. Continued thermal stimulation promotes secondary polymerisation, increasing polymer conversion and cross-linking density, which improves surface resistance to structural loss.

For subtractive monolithic materials, especially the polymer-infiltrated ceramic network (PICN) of Vita Enamic, the significant reduction in volumetric loss after ageing indicates a tribological shifting mechanism. Hydrothermal fatigue causes superficial microfissuring and particle debonding, as shown by AFM scans. During wear testing, these detached ceramic and filler particles remain in the contact zone, consistent with the larger defect radius measured in ImageJ. These particles form a ‘third-body’ protective layer or low-shear tribofilm, shielding the underlying material from deep vertical ploughing (H decreased from 0.05 ± 0.40 mm to 0.021 ± 0.06 mm for Vita Enamic). This mechanism explains the reduced macroscopic volume loss despite the disrupted local surface topography observed by AFM. The unpaired *t*-test revealed distinct, material-specific responses to hydrothermal and mechanical stress. For Saremco, isolated thermocycling did not significantly affect baseline maximum roughness (SC vs. STC, *p* = 0.1878). In contrast, mechanical wear with a zirconia ball produced a significant polishing effect (SC vs. SW, *p* = 0.0105). Voco showed significant surface profile degradation after isolated thermocycling, indicated by an increase in Sy values (VC vs. VTC, *p* = 0.0084). This suggests that the thermal expansion mismatch between the inorganic phase and polymer matrix generates micro-stresses at polymer–filler junctions. For Vita Enamic, hydrothermal ageing unexpectedly refined the surface, significantly reducing maximum profile height compared to the post-milling baseline (*p* = 0.0003). Unpaired *t*-tests across brands showed that mechanical function acts as a universal surface homogeniser. Although the materials initially had significantly different baseline topographies, with Voco smoother than Saremco (*p* = 0.0180) and Vita Enamic (*p* = 0.0009), these differences disappeared after physical wear. No significant differences were observed between material networks under isolated zirconia wear (SW vs. VW, *p* = 0.2856; SW vs. VTW, *p* = 0.2383) or the combined regime (S-W-TC vs. V-W-TC, *p* = 0.1822; S-W-TC vs. VI-W-TC, *p* = 0.2543). This statistical uniformity indicates that clinical wear against a smooth ceramic antagonist overrides initial material processing and composition, resulting in all modern restorative classes achieving a comparable, biofilm-resistant surface plateau.

Atomic force microscopy (AFM) analysis following thermal ageing indicates that CAD/CAM interim materials are vulnerable to hydrolytic and thermal stresses [25,26]. Control groups exhibit a smooth, isotropic surface texture due to high-pressure industrial manufacturing, which increases the polymer’s cross-linking density [3]. Prolonged thermal cycling causes a mismatch in thermal expansion between the organic matrix and inorganic fillers, leading to localised micro-separations [27,28,29]. This explains the pronounced microfissures in Group VIII (Tetric), where hydrolytic degradation occurs at the silane interface [30,31]. In contrast, the dual-network structure of Vita Enamic (Group VII) reduces the risk of large-scale structural failure. Its ceramic framework supports and confines the polymer domains under hydrothermal stress, maintaining stable, plateau-like regions [32,33]. Subtractively manufactured hybrid ceramics (Vita Enamic: 2.07 ± 0.03 mm^3^) exhibit high initial volumetric wear, leading to early material loss when used against rigid antagonists. A reduction in volumetric wear was observed for certain groups after thermocycling, contrary to standard expectations for hydrolytic ageing. This outcome is likely due to the thermal post-curing effect. Elevated temperatures during thermocycling appear to enhance monomer conversion and cross-linking in the polymer networks, increase surface hardness, and relieve residual polymerisation stresses. Together, these factors offset early hydrolytic degradation and improve wear resistance during the initial simulated mastication phase.

However, the formation of a protective, low-shear tribofilm after ageing stabilises their volume. As a result, milled monolithic blocks are suitable for long-term interim restorations or complex full-mouth rehabilitations where maintaining vertical dimension stability is essential. The additively manufactured groups (Voco and Saremco) showed low volumetric wear rates before and after ageing, challenging the view that printed polymers are inferior to industrially polymerised blocks. For short- to medium-term use, including crowns and bridges lasting up to six months, modern 3D-printed resins provide a reliable, cost-effective, and mechanically stable option. A significant decrease in wear parameters after ageing, confirmed by independent *t*-tests, shows that intraoral temperature changes from daily food and drink do not accelerate macro-structural failure. In 3D-printed materials, this thermal energy serves as an in vivo post-curing step, enhancing polymer conversion and cross-linking in the oral environment. This process reduces the risks linked to minor chairside undercuring [34,35]. All material groups were statistically equivalent after mechanical wear (*p* > 0.05 across all inter-brand comparisons), which is reassuring from a biological perspective. Regardless of initial surface roughness or manufacturing method, mastication against a smooth antagonist produces a similarly polished surface in all restorative materials. Clinically, this suggests that all tested materials will ultimately offer comparable resistance to bacterial biofilm and plaque, reducing the risk of localised periodontal inflammation or secondary caries at restoration margins [36,37]. The clinical longevity of dental restorative materials depends on their surface mechanical properties and ability to withstand the oral environment. This study assessed the Vickers microhardness of four dental materials before and after artificial thermal ageing (thermocycling) to evaluate structural resistance and degradation [38]. Results showed significant variation in baseline values and material-specific responses to thermal stress. VITA ENAMIC demonstrated a significantly higher baseline microhardness than all tested resin-based composites due to its unique polymer-infiltrated ceramic network (PICN) structure. This material features a porous feldspathic ceramic scaffold infiltrated with a polymer framework, forming a continuous ceramic backbone that offers greater indentation resistance than traditional hybrid composites, which use a polymer matrix with dispersed inorganic fillers. Thermocycling simulates the thermal and hydrolytic stresses experienced in vivo. Alternating temperatures cause expansion and contraction, creating internal stresses due to differences in thermal expansion between the resin and filler particles. This process explains the significant reductions in microhardness observed in VITA ENAMIC, TETRIC, and VOCO. For TETRIC and VOCO, water absorption during thermal cycling acts as a plasticiser, degrading silane coupling agents and polymer chains, which leads to surface softening and structural degradation. VITA ENAMIC also showed a notable microhardness reduction (approximately 12.2%), but its post-ageing value remained substantially higher than the baseline values of any composite, indicating strong long-term mechanical stability. SAREMCO had the lowest baseline hardness but remained stable after thermal ageing, with no statistically significant change. This stability may result from an optimised monomer composition or efficient cross-linking that limits water uptake and swelling. Although its low hardness restricts its use to areas with low masticatory stress, its chemical resistance to hydrolytic degradation is notable.

In contrast, VOCO showed a significant decrease in microhardness after thermocycling, indicating high susceptibility to water-induced degradation. The final microhardness of VOCO was similar to that of SAREMCO, as shown by overlapping standard deviation error bars.

Milled blocks demonstrate superior performance compared to 3D-printed resins because their dense, industrial-grade structure provides greater resistance to hydrothermal swelling and interlayer delamination than the heterogeneous, layer-by-layer structure of printed alternatives [39]. In particular, CAD/CAM materials such as polymer-infiltrated ceramic networks prevent polymer domain collapse under thermal stress, while 3D-printed resins experience accelerated softening and structural degradation when subjected to combined wear and ageing [40,41]. The laboratory findings provide valuable insights into the clinical performance of digital restorative materials. Industrially manufactured CAD/CAM materials, such as Vita Enamic and Tetric CAD, showed greater stability in nanoroughness and microhardness than 3D-printed resins like Saremco and Voco V-Print c&b temp. Lower post-wear nanoroughness reduces bacterial adherence and biofilm maturation, decreasing the risk of secondary caries and marginal staining. Higher microhardness helps preserve occlusal anatomical landmarks and proximal contacts. While the tested 3D-printed polymers offer promising mechanical properties for provisional or medium-term use, high-density milled frameworks remain more reliable for long-term, load-bearing restorations where surface integrity and gloss must be maintained. The growth of additive manufacturing in restorative dentistry reflects a wider move toward digital fabrication. In addition to standard 3D printing of interim polymer frameworks, advanced multi-material processing and structural programmability are transforming component design across engineering sectors [42]. Recent advances in material reinforcement, such as adding multi-walled carbon nanotubes and specialised fillers to thermoplastic matrices, have greatly improved the mechanical properties and stability of printed polymers [43]. Together, these cross-disciplinary developments show that additive manufacturing can address traditional structural challenges and offer scalable solutions for complex wear environments [44]. Integrating multi-walled carbon nanotubes and specialized fillers into thermoplastic matrices has significantly improved the mechanical properties and structural stability of printed polymers [42]. These advances show that additive manufacturing can overcome traditional structural limitations and provide scalable solutions for demanding wear environments. Although the combined ageing protocol reduced surface qualities across all materials, printed polymers demonstrated consistent volumetric stability. The additively manufactured groups, Voco and Saremco, exhibited low volumetric wear rates both before and after ageing. These findings challenge the conventional perspective that printed polymers are inferior to industrially polymerised blocks. Consequently, for short- to medium-term applications such as crowns and bridges with durations up to six months, contemporary 3D-printed resins provide a reliable, cost-effective, and mechanically stable alternative. This in vitro study identified distinct degradation pathways in additive and subtractive polymers; however, several limitations remain. The laboratory ageing protocols, although rigorously sequential, do not fully replicate the complex biochemical environment of the oral cavity, including salivary enzymes, pH variability, and diverse masticatory forces. Additionally, atomic force microscopy (AFM) assessments were confined to specific localised scan zones, potentially overlooking larger macrostructural defects. Long-term clinical trials are required to validate these submicron topographical and microhardness findings in vivo.

## 5. Conclusions

Within the limitations of this in vitro study, the following clinical implications can be drawn:

1. Simulated oral ageing reveals a distinct tribological divergence, where milled CAD/CAM blocks demonstrate superior structural resistance compared to 3D-printed resins, which suffer from interlayer heterogeneity.
2. The two additive manufacturing materials display distinct thermal behaviours. Saremco maintains polymer matrix stability at different temperature variations, whereas Voco is prone to rapid hydrothermal swelling and degradation.
3. Subjected to concurrent mechanical wear and thermal ageing, Vita Enamic maintains its structural integrity due to its rigid ceramic matrix. In contrast, Tetric exhibits severe filler debonding, and 3D-printed resins show rapid interlayer delamination.

## Figures and Tables

**Table 1:** Composite resins included in the study.

Material	Type	Manufacturer	Filler	Monomer	Shade/Translucency
Vita Enamic (V)	Hybrid ceramic	VITA Zahnfabrik, Bad Säckingen, Germany	Feldspar ceramic enriched with aluminium oxide 86%	UDMA and TEGDMA	A2/MT
Tetric CAD (T)	Composite resin	Ivoclar Vivadent, Liechtenstein	Nano-filled 70% with barium glass and silicon dioxide	Bis-GMA, Bis-EMA, TEGDMA, and UDMA	A2
Saremco Crowntec	Composite resin	SAREMCO Dental AG, Rebstein, Switzerland	Silanised Dental Glass combined with Pyrogenic Silica,	Methacrylic Acid Esters (Ethoxylated	A2
Voco V-Print c&b temp	Composite resin	VOCO GmbH, Cuxhaven, Germany	Silicon Dioxide (Silica) Nanoparticles, Barium–Aluminium–Borosilicate Glass Particles	TEGDMA (Triethylene glycol dimethacrylate)	A2

**Table 2:** Mean and standard deviation values for V (volumetric loss), height of the vertical loss (h), specific wear rate (SWR), and wear rate (WR) for the tested samples, printed and milled.

Material Group	V (mm^3^)	H (mm)	SWR ×10^−3^ mm^3^/(N·m)	WR (μm/1000 Cycles)
Voco	0.68 ± 0.01	0.029 ± 0.01	0.40 ± 0.03	3.98 ± 0.40
Saremco	0.43 ± 0.20	0.019 ± 0.30	0.30 ± 0.06	3.30 ± 0.26
Vita Enamic	2.07 ± 0.03	0.05 ± 0.40	1.06 ± 0.35	5.6 ± 0.40
Tetric	0.48 ± 0.015	0.054 ± 0.23	0.37 ± 0.023	4.31 ± 0.16
Voco after thermal ageing	0.17 ± 0.40	0.014 ± 0.12	0.13 ± 0.02	2.45 ± 0.06
Saremco after thermal ageing	0.188 ± 0.15	0.015 ± 0.25	0.14 ± 0.03	2.55 ± 0.50
Vita Enamic after thermal ageing	0.54 ± 0.30	0.021 ± 0.06	0.35 ± 0.08	3.59 ± 0.30
Tetric after thermal ageing	0.24 ± 0.30	0.016 ± 0.02	0.17 ± 0.25	2.89 ± 0.40

**Table 3:** Mean and standard deviation values for the nanoroughness parameter (Sa).

	VOCO	SAREMCO	VITA ENAMIC	TETRIC
Sa C	11.22 ± 1.83	18.12 ± 5.8	30.19 ± 6.59	20.24 ± 4.08
SaTC	13.45 ± 2.77	18.50 ± 6.17	11.02 ± 2.20	20.47 ± 2.94
Sa W	9.12 ± 2.09	9.91 ± 2.75	8.89 ± 1.09	10.28 ± 2.20
Sa W + TC	4.14 ± 0.86	4.95 ± 1.63	5.5 ± 1.28	5.44 ± 1.36

**Table 4:** Statistical analysis results after *t*-test (*p* ≤ 0.05). C—control; TC—thermal ageing; W—wear; S—Saremco; V—Voco; Vi—Vita Enamic; Te—Tetric.

Type of Material	*p* Values
SC-TC	0.1878914
SC-SW	0.0105795
SC-SW-TC	0.0004762
STC-STC W	0.0686249
SC-VC	0.0180795
SC-VIC	0.0009329
SC-TEC	0.1704765
STC-VTC	0.1252426
STC-VITC	0.1095338
STC-TETC	0.2130257
SW-VW	0.2856943
SW-VTW	0.2383789
SW-TESW	0.4141787
S-W-TC-V-W-TC	0.1822776
S-W-TC-VI-W-TC	0.2543384
S-W-TC-TE-W-TC	0.3108248
VC-TC	0.0084862
VC-WEAR	0.0752688
VC-W-TC	0.000149
VTC-TC WEAR	0.0003035
C-TC	0.0003742
C-WEAR	0.0001972
C-W-TC	0.0001972
TC-TC WEAR	0.0011274
C-TC	0.4635424
TC-TW	0.0022033
TTC-TW-TC	7.501 × 10^−5^
TC-TC WEAR	2.104 × 10^−5^

**Table 5:** Mean and standard deviation values for the nanoroughness parameter Sy. C—control; TC—thermal ageing; W—wear; W + TC—wear and thermal ageing.

	S	V	VI	T
Sy C	170.89 ± 73.98	86.92 ± 25.16	253.61 ± 44.37	158.23 ± 34.20
SyTC	153.69 ± 66.16	114.15 ± 42.62	99.90 ± 17.08	141.64 ± 29.78
Sy W	78.05 ± 21.98	69.41 ± 12.39	76.52 ± 16.72	85.51 ± 20.70
Sy W + TC	45.52 ± 16.89	34.80 ± 8.67	48.01 ± 18.48	49.51 ± 14.20

**Table 6:** Statistical results between the samples before and after thermal ageing.

Type of Material	Control Mean (± SD) [N/mm^2^]	TC Mean (± SD) [N/mm^2^]	*t*-Statistic	
VOCO	34.28 ± 0.94	28.32 ± 1.04	9.51	<0.001
SAREMCO	26.98 ± 0.25	26.60 ± 0.89	0.923	0.383
VITA ENAMIC	184.20 ± 6.42	161.60 ± 3.97	6.694	<0.001
TETRIC	68.18 ± 2.41	63.40 ± 3.21	2.664	0.029

## Data Availability

The raw data supporting the conclusions of this article will be madeavailable by the authors on request
